# Human T cell responses to Dengue and Zika virus infection compared to Dengue/Zika coinfection

**DOI:** 10.1002/iid3.203

**Published:** 2017-12-28

**Authors:** Jessica Badolato‐Corrêa, Juan Camilo Sánchez‐Arcila, Thiara Manuele Alves de Souza, Luciana Santos Barbosa, Priscila Conrado Guerra Nunes, Monique da Rocha Queiroz Lima, Mariana Gandini, Ana Maria Bispo de Filippis, Rivaldo Venâncio da Cunha, Elzinandes Leal de Azeredo, Luzia Maria de‐Oliveira‐Pinto

**Affiliations:** ^1^ Laboratory of Viral Immunology Fundação Oswaldo Cruz Instituto Oswaldo Cruz Rio de Janeiro Brazil; ^2^ Laboratory of Genetics, Institute of Paediatrics and Puericulture Martagão Gesteira (IPPMG) Federal University of Rio de Janeiro, UFRJ Rio de Janeiro Brazil; ^3^ Laboratory of Cellular Microbiology Fundação Oswaldo Cruz Instituto Oswaldo Cruz Rio de Janeiro Brazil; ^4^ Laboratory of Flavivirus, Fundação Oswaldo Cruz Instituto Oswaldo Cruz Rio de Janeiro Brazil; ^5^ Department of Clinical Medicine Universidade Federal do Mato Grosso do Sul Brazil; ^6^ Fundação Oswaldo Cruz Campo Grande Mato Grosso do Sul Brazil

**Keywords:** Dengue, Zika: T lymphocytes

## Abstract

**Introduction:**

Zika virus (ZIKV) and dengue virus (DENV) co‐circulated during latest outbreaks in Brazil, hence, it is important to evaluate the host cross‐reactive immune responses to these viruses. So far, little is known about human T cell responses to ZIKV and no reports detail adaptive immune responses during DENV/ZIKV coinfection.

**Methods:**

Here, we studied T cells responses in well‐characterized groups of DENV, ZIKV, or DENV/ZIKV infected patients and DENV‐exposed healthy donors. We evaluated chemokine receptors expression and single/multifunctional frequencies of IFNγ, TNF, and IL2‐producing T cells during these infections. Even without antigenic stimulation, it was possible to detect chemokine receptors and IFNγ, TNF, and IL2‐producing T cells from all individuals by flow cytometry. Additionally, PBMCs’ IFNγ response to DENV NS1 protein and to polyclonal stimuli was evaluated by ELISPOT.

**Results:**

DENV and ZIKV infections and DENV/ZIKV coinfections similarly induced expression of CCR5, CX3CR1, and CXCR3 on CD4 and CD8 T cells. DENV/ZIKV coinfection decreased the ability of CD4^+^ T cells to produce IFNγ^+^, TNF^+^, TNF ^+ ^IFNγ^+^, and TNF ^+^ IL2^+^, compared to DENV and ZIKV infections. A higher magnitude of IFNγ response to DENV NS1 was found in donors with a history of dengue infection, however, a hyporesponsiveness was found in acute DENV, ZIKV, or DENV/ZIKV infected patients, even previously infected with DENV.

**Conclusion:**

Therefore, we emphasize the potential impact of coinfection on the immune response from human hosts, mainly in areas where DENV and ZIKV cocirculate.

## Introduction

Dengue virus (DENV) and Zika virus (ZIKV) belong to Flaviviridae family and both diseases affect significantly human health. These viruses are mainly transmitted by *Aedes aegypti* or *albopictus* infected mosquitoes. Other routes of infection, including sexual, maternal, and blood transfusions, have been recently reported for ZIKV 1. DENV and ZIKV, like other flaviviruses, are single‐stranded, positive‐sense RNA viruses with a genome of 10.7 kb and two flanking non‐coding regions (5′ NCR and 3′ NCR). The open reading frame encodes one polyprotein with three structural proteins: capsid, pre‐membrane/membrane, and envelope and seven nonstructural proteins: NS1, NS2A, NS2B, NS3, NS4A, NS4B, and NS5 2. Four serotypes of DENV (DENV‐1 to −4), antigenically distinct have been described 3. In ZIKV, two major lineages, African (Nigeria, Senegal, and Uganda strains) and Asian (Malaysia 1966, Yap State 2007, and Cambodia 2010) have been described based on full genome sequences of the ORFs 4. The four DENV serotypes share approximately 70% amino acid identity with each other, while ZIKV displays an overall 43% homology with DENV (with up to 68% identity for more conserved non‐structural proteins) 1.

Dengue incidence has increased 30‐fold in the last five decades 5. Currently, dengue is endemic in 128 countries, most of them developing nations, affecting approximately 3.97 billion people annually 6. The incidence of dengue increased greatly over the past two decades in Brazil, affecting all regions of the country, except the South 7. Forty years after its discovery, ZIKV reemerged during a 2007 outbreak on Yap Island in Micronesia, continued in 2013 in French Polynesia 8, 9, 10, and in 2014 moved to multiple Pacific islands. At the end of that period, it was introduced to South America 11, 12, 13, 14, 15. In Brazil, the first reports of suspected cases occurred in the Northeast, with a peak during the first quarter of 2015, but it was only confirmed in April 2015. The epidemic continued spreading in May 2016. Since then, autochthonous transmission of ZIKV had been reported in 42 countries and territories in the Region of the Americas 11, 13, 16, 17. Due to lack of reliable official data, Brazilian Ministry of Health estimated the number of cases based on reports of attack rates from other countries.

Dengue infection may be asymptomatic or cause a febrile illness (dengue fever), accompanied by severe headaches, retro‐orbital pain, myalgia, arthralgia, gastro‐intestinal complications, liver inflammation, and skin rashes. When fever subside, patients may develop a more severe life‐threatening condition, characterized by an increase in vascular permeability, plasma leakage and hemorrhagic manifestations, leading to hypovolemic shock 18. Clinical features of ZIKV infection resemble—but are generally milder—those caused by DENV. It could range from asymptomatic infection to a febrile illness characterized by rash, fever, conjunctivitis, arthralgia, and arthritis 10, 14, 19. Unexpectedly, ZIKV outbreak also had a high attack rate and revealed an association with the appearance of Guillain‐Barré syndrome in adults 14, 20 and devastating congenital birth defects, including microcephaly in the developing fetus. It makes of Zika a major emerging public health problem 14, 21, 22.

T cells have an essential role in protection against a variety of infections. Indeed, the development of successful vaccine formulations will require the generation of potent and long‐lasting T‐cell responses. However, there are still no clearly defined immune correlates of protection for these infections 23. The role of T cells during dengue infection is still controversial, with studies supporting either an immunoprotective or immunopathological role (reviewed in 24). Pioneer studies proposed that T cells have a detrimental role during secondary dengue infections in a process termed “original antigenic sin.” Based on this theory, cross‐reactive T cells generated during primary infection, which recognize secondary‐infected DENV serotype with low affinity, are poorly functional but prone to inducing immunopathology 25. Thus, cross‐reactive memory T cells are present in increased numbers and have a low activation threshold. They may outcompete their naïve subsets that have high affinity for secondary‐infected serotype with an overall detrimental outcome for protective immunity 25. Collectively, studies showed that dengue infection elicits a broad specific T cell response that peaks around day 8–10 from fever onset 24, 26. Dengue‐specific CD8^+^ T cells are present at higher frequencies compared to their CD4^+^ counterparts and preferentially target non‐structural proteins NS3, NS4b, and NS5, while CD4^+^ T cells are mainly directed toward the capsid envelope and the secreted protein NS1 27. A comparison of amino acid sequences of DENV and ZIKV CD8^+^ T cell epitopes point out a high sequence homology between the two viruses and suggests that some of these CD8^+^ epitopes may also exist in ZIKV 24.

Therefore, we evaluated a cohort of well‐characterized DENV, ZIKV, or DENV/ZIKV‐infected patients and DENV‐exposed healthy donors. Molecular and serological methodologies confirmed all infections. Even without specific in vitro antigenic stimulation, DENV, ZIKV, and DENV/ZIKV infections induced expression of CCR5, CX3CR1, and CXCR3 on CD4^+^ and CD8^+^ T cells, indicating an activated status of these cells. However, DENV/ZIKV coinfection decreased the ability of CD4^+^ T cells to produce IFNγ^+^, TNF^+^, TNF^+^ IFNγ^+^, and TNF^+^ IL2^+^, compared to DENV and ZIKV infections. Finally, a hyporesponsiveness of effector/memory T cells in most of acute patients against DENV NS1 specific antigen might occur due to a clonal exhaustion. We would like to emphasize the potential impact of coinfection on the immune response from a human host.

## Materials and Methods

### Ethics statement

Human blood samples were obtained after written informed consent from all participants. The study was conducted in accordance to the project approved by the Ethics Committee of Plataforma Brasil, FIOCRUZ (CAAE 13318113 · 7 · 0000 · 5248).

### Human blood samples

Blood samples from dengue‐exposed individuals were obtained from discarded routine blood donations’ buffy coats at Clementino Fraga Filho University Hospital (Brazil) during 2013. Because these samples were collected anonymously, they were exempt from informed consent. All four healthy adult donors were seronegative for DENV IgM, seropositive for DENV IgG, negative for RT‐PCR ZIKV and with no clinical history of infections in the past 3 months, suggesting that donors had experienced at least one DENV infections prior to blood donation. Patients’ blood samples were collected between February and March 2016 in ACD Vacutainer and dry tubes. Physicians at Walfrido Arruda Emergency Care Unit, Coronel Antonino (Mato Grosso do Sul, Brazil) evaluated clinical parameters and classified all infected patients according to WHO, 2009 18. All samples were screened for DENV, ZIKV, and CHIKV as a differential diagnosis.

### Diagnosis

Serum samples were used for all diagnostic tests described below. For diagnosis of suspect dengue cases, DENV IgM Capture DxSelect™ (Focus Diagnostics, CA, USA) and Platelia™ Dengue NS1 Ag ELISA (BioRad Laboratories, CA, USA) were performed. Molecular detection and serotype typing were performed as previously described 28, by real‐time RT‐PCR protocol 29 and Simplexa™ Dengue Real Time RT‐PCR (Focus Diagnostics, Cypress, CA, USA) according to manufactureŕs protocol. We considered a positive diagnosis for DENV the samples positive for DENV qRT‐PCR or/and Dengue NS1 Ag, as stated above. Dengue patients were considered with primary infection provided being positive for IgM, whether negative for IgG or positive for IgG, provided the rate IgM/IgG was greater than 2.0. Dengue cases considered secondary infection presented IgM/IgG rate less than 2.0 18. We considered a positive diagnosis for Zika the samples positive for real‐time RT‐PCR for ZIKV, as described previously 19. DENV/ZIKV coinfected patients presented both criteria mentioned above for DENV and Zika positivity. For diagnosis of the suspected chikungunya cases, it was performed anti‐CHIKV IgM capture ELISA described by CDC 30 and Brazilian Ministry of Health 31, anti‐CHIKV ELISA IgM (Euroimmun, Lubeck, Germany) and molecular RT‐PCR protocol for CHIKV as described previously 32. Patient details are provided in Table 1.

**Table 1:** Characteristics of the patient's cohort used for this study

Patient code	Age (yr)	Gender	Type of infection^a^	Days of disease^b^	Severe DENV (WS/NWS)^c^	qRT‐PCR DENV	NS1 DENV	IgM DENV	IgM CHIKV	qRT‐PCR ZIKV	Platelets/mm^3^	Leukocytes/mm^3^	Lymphocytes/mm^3^
DENV													
1	39	Male	Secondary	4	No (NWS)	DENV‐1	pos	neg	neg	neg	248	4400	2464
2	47	Female	Secondary	22	No (WS)	DENV‐1^d^	neg	pos	neg	neg	117	–	–
3	69	Male	Secondary	3	No (WS)	DENV‐1	pos	pos	neg	neg	98	2200	–
4	27	Female	Secondary	2	No (NWS)	DENV‐4	neg	neg	neg	neg	209	7800	1248
5	52	Female	Primary	3	No (NWS)	DENV‐1	pos	neg	neg	neg	163	3400	272
6	30	Female	Secondary	6	No (NWS)	neg	pos	pos	neg	neg	172	2700	575
Median [IQR]	43 [29–56]			4 [3–10]							168 [112–219]	3400 [2450–6100]	912 [348–2160]
ZIKV													
7	38	Female	Secondary	5		neg	neg	neg	neg	pos	197	3900	1521
8	28	Male	Primary	4		neg	neg	neg	neg	pos	128	3800	1330
9	45	Male	Secondary	3		neg	neg	neg	neg	pos	246	4000	1640
10	60	Female	Secondary	4		neg	neg	neg	neg	pos	289	6100	3416
Median [IQR]	42 [31–56]			4 [3–5]							222 [145–278]	3950 [3825–5575]	1581 [1378–2972]
DENV/ZIKV													
11	14	Male	Primary	2	No (NWS)	neg	pos	neg	neg	pos	260	4600	2024
12	46	Male	Secondary	5	No (NWS)	neg	pos	neg	neg	pos	151	5300	2120
13	19	Female	Secondary	8	No (NWS)	DENV‐1	inconcl	neg	neg	pos	296	9900	2871
14	33	Female	Secondary	8	No (NWS)	DENV‐1	neg	neg	neg	pos	239	6600	1848
15	21	Female	Secondary	4	No (NWS)	DENV‐1	neg	neg	neg	pos	137	4700	2585
16	48	Male	Primary	3	No (NWS)	DENV‐4	pos	neg	neg	pos	147	3000	870
17	18	Male	Secondary	2	No (NWS)	DENV‐1	neg	pos	neg	pos	308	6000	780
Median [IQR]	21 [18–46]			4 [2–8]							239 [147–296]	5300 [4600–6600]	2024 [870–2585]

– Data not available.

^a^ Performed by the IgG DENV.

^b^ Days after the initial symptoms.

^c^ WS/NWS, dengue fever with warning signs/dengue fever without warning signs.

^d^ Patient 2 confirmed DENV‐1 infection using Simplexa™ Dengue Real Time RT‐PCR (Focus Diagnostics, Cypress, CA) (CT = 34) even after 22 days of onset of symptoms.

### Reagents, proteins, and monoclonal antibodies

A mammalian recombinant DENV Non‐Structural‐1 (DENV NS1) from all four serotypes was used as stimuli for ELISPOT. This protein was donated by The Native Antigen Company (https://thenativeantigencompany.com/product/dengue-virus-ns1-protein-serotypes-1-4/?doing_wp_cron=1480736436.0465950965881347656250). Phytohemagglutinin (PHA), phorbol 12‐myristate 13‐acetate (PMA), ionomycin, Brefeldin A, and Saponin were supplied by Sigma–Aldrich (St. Louis, MO, USA). Detailed information of all mAbs used in this study is listed in Table S1.

### PBMC isolation and culture

Briefly, peripheral blood mononuclear cells (PBMCs) and plasma were isolated by Ficoll‐Paque™ PLUS density gradient centrifugation (GE Healthcare, Uppsala, Sweden) and frozen in fetal bovine serum (FBS, Gibco, Invitrogen Co, Carlsbad, CA, USA) supplemented with 10% dimethyl sulfoxide (DMSO, Sigma–Aldrich). Cells were thawed on the day of the experiment and were used directly for ex vivo assay as follows.

### Extracellular staining

Cells were stained for surface markers (FITC anti‐CD3, APCCy7 anti‐CD4, AmCyan anti‐CD8, PECy7 anti‐CX3CR1, Pacific Blue anti‐CCR5 and, PerCP anti‐CXCR3) (Table S1) for 30 min, then washed, fixed with 2% paraformaldehyde, and maintained in PBS. The data were collected using BD® FACS ARIA IIu flow cytometer and analyzed using FlowJo 10 software (Tree Star®).

### Intracellular staining (ICS)

For intracellular cytokine staining (ICS), PBMCs (2 × 10^5^ cells/well) were incubated without stimuli or with PMA (10 ng/mL)/Ionomycin (1 μg/mL) for 2 h at 37°C. Then, Brefeldin A (10 μg/mL) was added to the cultures and incubated for 4 h. Cells were then washed and stained for extracellular markers for 30 min using BV510 anti‐CD3, PECy7 anti‐CD4, and PETexasRed anti‐CD8. After that, cells were washed and fixed with 2% paraformaldehyde. For ICS, cells were blocked with bovine serum albumin (1% BSA, Sigma–Aldrich), permeabilized with saponin (0.05%) and stained with eFluor® 660 anti‐IFNγ, Alexafluor® 700 anti‐TNF, and eFluor® 450 anti‐IL2 (Table S1). Samples were analyzed on BD® FACS ARIA IIu flow cytometer and analyzed using FlowJo 10 software (Tree Star®).

### Ex vivo IFNγ ELISPOT assay

IFNγ enzyme‐linked immunosorbent spot (ELISPOT) were performed using DENV‐exposed donors’ or acute patients’ thawed PBMCs that were immediately added to ELISPOT plates. Briefly, 96‐well plates (Multiscreen HTS; Millipore, Burlington, MA, USA) were coated overnight at 4°C with 2.5 μg/mL of capture mouse anti‐human IFNγ antibody (clone 1‐DK1; Mabtech, Nacka Strand, Sweden). The plates were washed with phosphate‐buffered saline (PBS) and blocked with RPMI 1640 (Gibco, Invitrogen Co) supplemented with 10% heat‐inactivated FBS for 1 h at room temperature. Blocking solution was removed, and 2 × 10^5^ PBMCs were plated per well in the presence or absence of NS1 protein from all four DENV serotypes at a concentration of 0.1 μg/mL. After 20–24 h of incubation plates were washed and 1 μg/mL of biotinylated anti‐human IFNγ (clone 7‐B6‐1; Mabtech) was added for 2 h at room temperature. After washing, 100 μL of streptavidin‐alkaline phosphatase (Mabtech) was added and the plates were incubated in the dark for 1 h at room temperature. Then, plates were washed, and 50 μL of alkaline‐phosphatase substrate 5‐bromo‐4‐chloro‐3‐indolyl‐phosphate/nitro blue tetrazolium chloride (BCIP‐NBT); KPL (Gaithersburg, MD, USA) was added. After 10–15 min, colorimetric reaction was stopped with running tap water. Spots were counted using an automated ELISPOT reader (ImmunoSpot® S6UV Ultra, Cleveland, OH, USA). The number of IFNγ‐producing cells was expressed as spot‐forming cells (SFC) relative to 10^6^ PBMCs. Values were calculated by subtracting the number of spots detected in unstimulated control wells. Values were considered positive if they were equal or greater than 10 spots and at least two times above the mean of unstimulated control wells. As a positive control, cells were stimulated with phytohemagglutinin (PHA at 5 μg/mL).

### Statistics

ELISPOT and chemokines receptors analysis were determined using nonparametric two‐tailed Mann–Whitney (Graph Pad Prism ver. 5.0). Multifunctional analysis of cytokine frequencies was performed using Boolean gating in FlowJoX ver. 10.3 and GraphPad Prism ver. 6.0. To compare the frequency of the multifunctional populations among the groups Mann–Whitney test was used. The differences of variables among groups were considered significant when *p* < 0.05.

## Results

### Characteristics of patient's cohort

Peripheral venous blood was obtained from 4 DENV‐acute patients and 1 late‐acute phase disease (with 22 days’ illness), 4 ZIKV‐acute patients and, 7 DENV/ZIKV coinfected acute patients. As shown in Table 1, patient 2 confirmed DENV‐1 infection using Simplexa™ Dengue Real Time RT‐PCR (Ct, Cycle threshold value = 34.6) even after 22 days of onset of symptoms. All four matched healthy donors were negative for DENV IgM, positive for DENV IgG, and negative for RT‐PCR ZIKV, so they were referred here as DENV‐exposed donors. Thirteen patients (76.5%) had dengue during their lifetime, presumed by the positivity for DENV IgG, in which 5 individuals were from DENV group, 3 from ZIKV, and 5 from DENV/ZIKV coinfection group. Most of DENV‐ or DENV/ZIKV patients showed a mild clinical form (non‐warning signals or NWS) and no fatal cases were observed among the cohort studied. In general, symptoms and signs caused by these viruses were similar among the studied groups, and the patients presented typical symptoms such as fever, rash, arthralgia, myalgia, fatigue, headache, and conjunctival hyperemia. No differences were observed in age, gender distribution or days of disease comparing all groups. Similarly, no differences were seen for platelets, leukocytes, and lymphocytes counts among groups. The serotype DENV‐1 was predominant among DENV‐ or DENV/ZIKV‐coinfected patients (80%). Finally, all studied samples were also negative for CHIKV diagnosis. The characteristics of all acute patients are detailed in Table 1.

### DENV, ZIKV, and DENV/ZIKV infection induce an increase of T CD4^+^ and CD8^+^ chemokine expression

**Figure 1:**
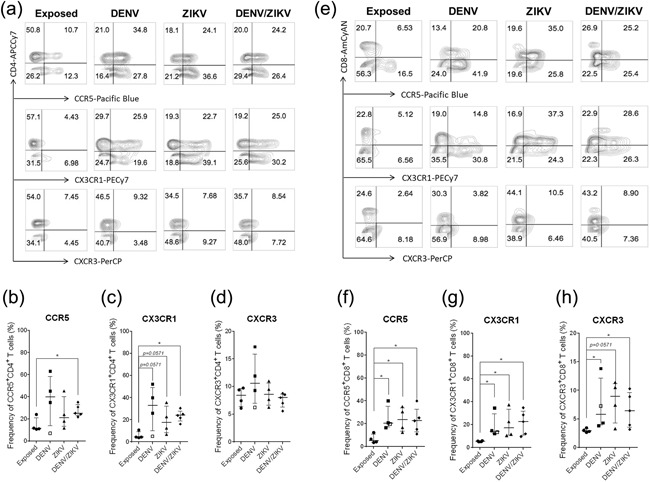
Chemokine receptor expression on CD4^+^ and CD8^+^ T cell populations in acute DENV, ZIKV, and DENV/ZIKV patients. Peripheral blood mononuclear cells (2 × 10^5^) were stained with mAb against surface markers CD3, CD8, CD4, CCR5, CX3CR1, and CXCR3. The expression of CCR5, CX3CR1, and CXCR3 on CD4^+^ (a) and CD8^+^ T cells (e) was showed in contour plots from one representative exposed donor, DENV‐, ZIKV, and DENV/ZIKV‐patients by flow cytometry. Frequency, median, 25th and 75th percentile of CCR5^+^ (b), CX3CR1^+^ (c), and CXCR3^+^ (d) CD4^+^ T cells from acute viral patients were compared between them and with those in exposed healthy controls. The same strategy was used for CD8^+^ T cells (f–h). Patient 2 in late acute phase (22 days of illness) was show as open squares. Statistical significance of differences between groups was determined by using two‐tailed Mann–Whitney test, where *p *< 0.05 were considered significant (*p < 0.05; **p < 0.01).

Chemokines and their receptors are key drivers of inflammation. Our goal here was to assess the magnitude of T cell activation by ex vivo expression of chemokine receptors, particularly useful for dissecting T‐cell subsets with distinct migratory capacity and effector function. As shown in Figure 1, expression of ex vivo chemokine receptors such as CCR5, CX3CR1, and CXCR3 were consistently detected on CD4^+^ and CD8^+^ T cells from all acute patients and healthy exposed donors. DENV/ZIKV infected individuals presented higher frequencies of CCR5^+^ or CX3CR1^+^ compared to exposed donors. Although not significant (*p* = 0.0571), we observed that DENV or ZIKV infected individuals have a propensity to have high CX3CR1^+^ frequencies compared to exposed individuals. (Fig. 1b,c). No appreciable differences were detected in CXCR3 expression on CD4^+^ T cells among all groups (Fig. 1d). Intriguingly, among patients analyzed in DENV group, the lowest values for all chemokine receptors expression on CD4^+^ T cells were observed for patient 2, in which cells were obtained 22 days post infection, even though viral genome was still detected.

CCR5, CX3CR1, and CXCR3 were detected in higher numbers of circulating CD8^+^ T cells in all three groups of infected patients in comparison to those exposed donors (Fig. 1f–h).

Therefore, regardless of acute viral infection, CD4^+^ and CD8^+^ T cells would be readily able to migrate and perform effector function.

Frequency of cytokine‐producing T CD4^+^ and CD8^+^ are differentially regulated in DENV, ZIKV, and DENV/ZIKV infection.

To assess the effector function of T cells by means of cytokine produced, we also evaluated the frequency of cells spontaneously producing TNF, IL2, and IFNγ.

**Figure 2:**
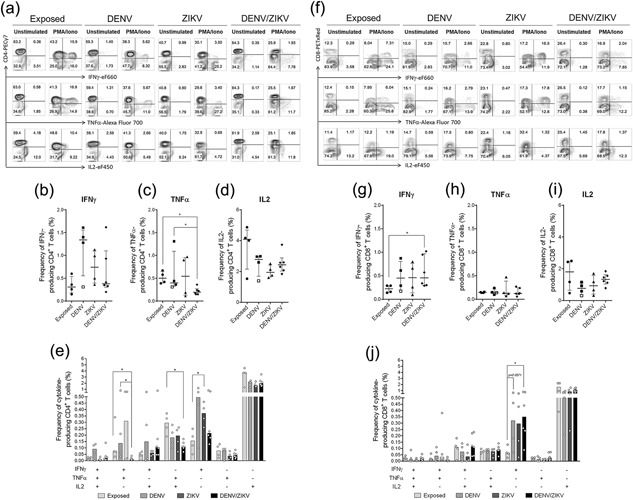
Frequency of IFNγ‐, TNF‐, and IL2‐producing CD4^+^ and CD8^+^ T cells in acute DENV, ZIKV, and DENV/ZIKV‐patients and exposed donors. Cultures of 2 × 10^5^ PBMCs were stimulated with PMA/Ionomycin or unstimulated (medium) for 6 h in presence of brefeldin in the last 4 h. Then, cells were stained with mAb against surface markers CD3, CD8, CD4, and mAb against intracellular IFNγ, TNF, and IL2. Frequency of IFNγ, TNF, and IL2 on CD4^+^ (a) and CD8^+^ T cells (f) was exhibited in counter plots from one representative exposed donor, DENV‐, ZIKV, and DENV/ZIKV‐patients by flow cytometry for both conditions. Frequency, median, 25th and 75th percentile of IFNγ^+^ (b), TNF^+^ (c), and IL2^+^ (d) CD4^+^ T cells in unstimulated condition from acute viral patients were compared between them and with those in exposed healthy controls. The same strategy was used (g–i). Patient 2 in late‐acute phase (22 days of illness) was show as open squares. Bars represent the median of frequency of CD4^+^ T cells (e) and CD8^+^ T cells (j) expressing each of the seven possible combinations of IFNγ, TNF, and IL2 among the studied groups in unstimulated condition. Statistical significance of differences between groups and comparisons among the multifunctional populations were determined by using two‐tailed Mann–Whitney test and represented by lines. Values of *p *< 0.05 were considered significant (*p < 0.05; **p < 0.01).

As shown in Figure 2, even without antigenic in vitro stimulation, we observed low, but detectable, frequencies of IFNγ^+^ (0.1–1.6%, minimum to maximum), TNF^+^ (0.06–1.3%) and IL2^+^ (0.3–1.8%) producing T cells from acute patients and in healthy DENV‐exposed donors’ T cells (IFNγ, 0.1–0.6%; TNF, 0.1–0.7%; IL2, 0.5–4.9%).

Initially, we evaluated separately the frequency of IFNγ, TNF, IL2 producer CD4^+^ and CD8^+^ T lymphocytes, regardless of their simultaneous production (Fig. 2b–d, g–i). First, we observed a trend toward an increased frequency of total IFNγ^+^CD4^+^ T cells in DENV (exception of patient 2, open squares) compared to exposed donors. DENV/ZIKV‐patients and exposed healthy donors had similar frequencies of total IFNγ^+^CD4^+^ T cells (Fig. 2b). Exposed donors, DENV‐ and ZIKV‐patients had a similar frequency of total TNF^+^CD4^+^ T cells, while DENV/ZIKV‐patients had significantly decreased frequencies of total TNF^+^CD4^+^ T cells compared to exposed donors and DENV‐patients (Fig. 2c). A trend toward decreased frequencies of total IL2^+^CD4^+^ T cells was seen in all acute patients compared to exposed donors (Fig. 2d).

Then, we evaluated the fractions of each multifunctional cell population expressing all three, any combination of two or single production of cytokines (Fig. 2e and j). Among T CD4^+^ lymphocytes, the least prevalent populations with two functions were IFNγ^+^TNF^+^ and IL2^+^TNF^+^ in DENV/ZIKV‐patients compared to exposed donors. Higher frequency of IFNγ^+^TNF^+^ CD4^+^ T cells was observed in DENV‐ compared to DENV/ZIKV‐patients. Finally, we observed increased frequency of single IFNγ^+^CD4^+^ T cell population in ZIKV‐patients compared to exposed donors (Fig. 2e).

A similar analysis was applied to CD8^+^ T cells. We detected a significant increase in total IFNγ^+^CD8^+^ T cells frequency in DENV/ZIKV‐patients compared to exposed healthy donors. A trend toward increased frequency of total IFNγ^+^CD8^+^ T cells was observed in ZIKV and in DENV patients (except for patient 2, open squares) (Fig. 2g). Exposed donors, DENV, ZIKV, and DENV/ZIKV‐patients had similar frequency of total TNF^+^CD8^+^ T cells (Fig. 2h). A decreased frequency trend of total IL2^+^CD8^+^ T cells, similarly to total IL2^+^CD4^+^ T cells, was detected in all acute patients compared to exposed donors (Fig. 2i). Regarding the multifunctional analysis, we only detected a higher frequency of single IFNγ^+^CD8^+^ T cells in DENV‐ and DENV/ZIKV‐patients compared to exposed donors (Fig. 2e). Therefore, we suggest that DENV/ZIKV coinfection may influence differently CD4^+^ and CD8^+^ T cells responses, an effect mainly observed in the frequencies of CD4^+^IFNγ^+^TNF^+^, CD4^+^TNF^+^IL2^+^, and CD8^+^ total IFNγ^+^ populations.

### DENV‐specific response targeting NS1 proteins was rarely detectable in acute patients

**Figure 3:**
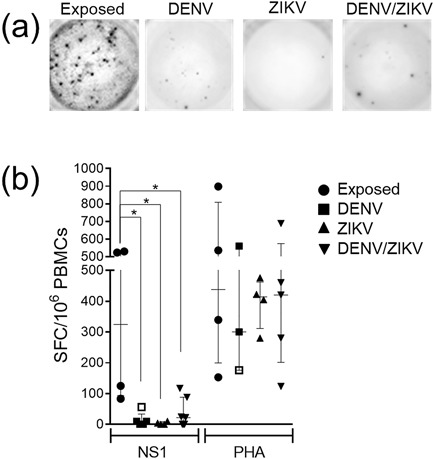
DENV‐specific cells targeting DENV 1–4 NS1 protein in acute DENV, ZIKV, and DENV/ZIKV‐patients, and donors experiencing dengue. Peripheral blood mononuclear cells (2 × 10^5^ in 0.1 mL) were incubated with recombinant mammalian (rm) NS1 (0.1 μg/mL) derived from four different DENV serotypes or PHA for 20 h and IFNγ production was measured by ELISPOT assay. (a) Representative IFNγ production from one representative exposed donor, DENV, ZIKV, and DENV/ZIKV‐patients are shown against rmNS1. (b) The values obtained of IFNγ production, expressed as spot‐forming cells (SFC) relative to 10^6^ PBMC, is shown for the rmNS1 and PHA were compared between them and with those in exposed healthy controls. Graphs show the median, 25th and 75th percentile from acute‐patients and DENV‐exposed ZIKV naïve donors. Statistical significance was determined by using the two‐tailed Mann–Whitney test, where *p *< 0.05 were considered significant (*p < 0.05; **p < 0.01).

We used DENV NS1 protein to evaluate DENV‐specific response in acute patients because ZIKV and DENV NS1 share 53–56% of amino acid identity 33. Moreover, NS1 has gained considerable attention for early dengue diagnostic tests. All healthy donors and 75% of acute patients in our cohort had indications of previous dengue, thus it was expected that they would be great responders of NS1 DENV. Considering this, we assessed DENV‐specific T cell response against a pool of NS1 from all four serotypes in PBMCs isolated from acute patients from DENV‐exposed donors by IFNγ ELISPOT assay (Fig. 3a).

Our initial objective was to compare NS1 DENV‐specific memory response from dengue‐exposed donors with dengue‐infected individuals. Our data indicate that DENV NS1 memory response had the highest frequencies and magnitude in healthy dengue‐exposed donors compared to those with active DENV infection. Among DENV ingle‐infected patients, the 22‐days DENV patient 2 showed the best response to NS1, indicating that latter phases of dengue could be a better time point for evaluating specific cell responses through ELISPOT. Our second goal was to determine whether active ZIKV infection could affect the NS1 DENV‐specific memory response in two different situations: in DENV‐exposed patients or in those who have current ZIKV or DENV/ZIKV infections. Our data showed that DENV NS1 was not able to induce a response in any acute active ZIKV‐patients, even the samples being positive for Dengue IgG ELISA test. Two out five DENV/ZIKV‐patients responded with above 50 IFNγ SFC following DENV NS1 stimulation, while the other three responded with below 20 IFNγ SFC after stimulation (Fig. 3b). Finally, the magnitude of IFNγ SFC response was not statistically different among the groups of acute patients. In order to understand the specificity of this response, we intend to evaluate healthy donors exposed to Zika in the future.

## Discussion

Although T lymphocytes are not infected by DENV and appear not to be infected by ZIKV 34, the impact of each one and of the DENV/ZIKV coinfection seem to affect their function. To address this investigation, we attempted to analyze some features of CD4^+^ and CD8^+^ T cell response in PBMC from patients in a cohort of DENV only, ZIKV only and DENV/ZIKV coinfected patients. Our work began with extensive fieldwork in the Midwest region of Brazil in 2016. There, 134 suspected cases of dengue or zika with an acute febrile illness were recruited, including those with by at least two of the signs and symptoms (headache, myalgia or arthralgia, rash, pruritus, retro‐orbital pain, and prostration). Methods of serology and molecular biology have been designed to diagnose the etiologic agent. Of the suspected cases, 6% of them confirmed ZIKV alone, 50% DENV, 22% were coinfected DENV/ZIKV, the others chikungunya, coinfections or not confirmed cases. At this point we realize that we did not have a larger number of samples with zika alone (Azeredo et al. data in submission). Therefore, we analyzed small numbers of individuals in each studied group to match ZIKV‐infected patients’ number, even though aware of the possible impact on dispersion of variables such as age, gender, and others because of a reduced number of donors. Despite that, we could report for the first time some aspects of DENV/ZIKV coinfection and compare then to DENV and ZIKV only infections, as well as healthy donors. Herein, our data reported from 17 well‐characterized patients infected by DENV, ZIKV or DENV/ZIKV and 4 healthy donors.

Our research evaluated the expression of chemokine receptors on T cells. Chemokine receptors have been useful for dissecting T‐cell subsets with distinct migratory capacity and effector function 35. We assessed the effector as well as memory T cells by an increased expression of CCR5, CX3CR1, or CXCR3, in contrast to their T naïve precursors. CCR5 and CXCR3 are closely linked to Th1 function on activated CD4^+^, memory/activated CD8^+^ T cells, and NK cells 36, 37, 38. Our data confirmed an increased expression of CCR5 on T cells from acute dengue patients 39, 40. CCR5 expression would be promoting an enhanced T cell recruitment into the liver, a hypothesis that was corroborated by a high frequency of CCL5^+^ cells in hepatic tissue from dengue fatal cases 40. It is widely accepted that CCR5 is part of host's immune response in the dengue, its role, mediating the traffic of immune cells from blood to target tissues or acting directly on the antiviral response, is still unknown. A recent study demonstrated CX3CR1‐based transcriptome and proteome‐profiling defined a core signature of memory CD8^+^ T cells with cytotoxic effector function 41. Highly polarized CX3CR1^+^ cytotoxic CD4^+^ T cells was specifically expanded in exposed donors and, in particular, in those donors carrying an HLA allele associated with protection from severe dengue 42. By the fact that an increased frequency of CD4 and CD8 T cells expressing CX3CR1 in patients with DENV and, for the first time, in patients with ZIKV and DENV/ZIKV, our data could indicate a potential cytotoxic capacity of T cells from these in acute patients. The protective role of CXCR3 against DENV infection has been demonstrated in experimental models since CXCR3^−/−^ mice infected by DENV presented a higher mortality rates than the wild‐type mice. Moreover, brains of CXCR3^−/−^ mice showed higher viral loads and quantitatively fewer CD8^+^ T cells than those of wild‐type mice 43. We hypothesize that an increase in the frequency CD4^+^ and CD8^+^ T cells expressing chemokine receptors would contribute to regulate virus progression through a precise control of inflammatory cells targeting the affected tissue. Thus, chemokine receptors would play a more immunoprotective role once T cells could exert an antiviral, effector, cytotoxic, and migratory activities.

The frequency of IFNγ‐producing T cells has been the most used parameter to assess an effective immune or vaccine‐induced response. Similarly to IFNγ, TNF is capable of mediating the killing of a variety of intracellular infectious viruses, bacteria, and parasites 44, 45. Although IL2 has little direct effector function, it promotes the expansion of CD4^+^ and CD8^+^ T cells, amplifying any potential effector T cell responses. Importantly, it has been demonstrated that frequency of cytokine‐producing T cells alone is not sufficient to predict protection. To provide prospective evidence of the quality of T cell response, vis‐à‐vis multifunctional T cells is required 23. Regarding dengue, compelling evidence of the importance of multifunctional T‐cells to mediate protection was found in Flavivirus‐naïve volunteers vaccinated with live, attenuated DENV‐1 vaccine, rDEN1Δ30. The authors observed that multifunctional T cells increased significantly in non‐viremic subjects tended to have a higher frequency of multifunctional T cells compared to viremic subjects. Therefore, the presence of multifunctional T cells following rDEN1Δ30 vaccine safely and effectively prompt immune responses associated with control of infection and protection from re‐infection 46.

Finally, we studied the magnitude of IFNγ response to DENV NS1. Gideon's team did not observe differences in IFNγ induction between peptide pools and recombinant proteins in overnight ELISPOT assay using PBMC from the same donors 47. A higher magnitude of IFNγ response to DENV NS1 was found in experienced dengue donors, but a hyporesponsiveness was found in any acute viruses, even experiencing dengue. In 2001, our group described a reduced frequency of CD4^+^ and CD8^+^ T lymphocytes and a poor ability for T‐lymphocyte proliferation in response to mitogens and dengue antigens in acute DENV‐infected patients, but re‐established in convalescence phase 48. Another study found that in vitro exposure to DENV‐2 of T lymphocytes isolated from healthy donors reduces the lymphoproliferative capacity in response to mitogen, suggesting that DENV‐2 can inhibit T cell‐mediated immunity, bypassing monocytes and dendritic cells, classical DENV cell targets 49.

Briefly, mono‐ and coinfections similarly induced expression of CCR5, CX3CR1, and CXCR3 on CD4^+^ and CD8^+^ T cells. This could promote functional and migratory similarities of these cells, regardless of the infecting virus. However, DENV/ZIKV coinfection decreased the ability of CD4^+^ T cells to produce IFNγ^+^, TNF^+^, TNF^+^IFNγ^+^, and TNF^+^IL2^+^, compared to monoinfections. We suppose two antagonistic scenarios: coinfected people are more immunocompromised than those monoinfected, so coinfection would be the worst scenario for those individuals. Another example of potential adverse effects of this immunosuppression is, that a reduced capacity to activate CD4^+^IFNγ^+^TNF^+^ and CD4^+^TNF^+^IL2^+^ T cell populations might interfere with future development of specific or cross‐reactive memory lymphocytes, leading to a weak response in a subsequent encounter to other potential arbovirus. In the other scenario, coinfected people have a less inflammatory milieu than monoinfected people. This could mean that a reduced inflammatory condition could avoid harmful effects like cytokine storm. Nevertheless, once the clinical outcome of the patients was similar, it was not possible to associate the symptoms and clinical signs with the immune profile of each group.

## Authors' Contributions

JBCdS, JCSA, ELdA, and LMdOP performed experiments, reviewed data, and planned the experimental strategy. MG collected data using a FACS ARIA BD flow cytometer. ABdP, JBCdS, TMAdSEP, LBdS, PCGN, and MdRQL performed all diagnostic tests. RVdC, JBCdS, TMAdS, LBdS, and ELdA collected samples and provided clinical information. JBCdS, JCSA, and LMdOP conceived and directed the study, and wrote the manuscript. All authors have critically read and edited the manuscript.

## Conflicts of Interest

The authors declare no conflicts of interest.

## Supporting information

Additional supporting information may be found in the online version of this article at the publisher's web‐site.

**Table S1**. Monoclonal antibodies used in this study.
