# Supplementary material for: Human T cell responses to Dengue and Zika virus infection compared to Dengue/Zika coinfection
Source: Immun Inflamm Dis. 2017 Dec 28;6(2):194–206. doi: 10.1002/iid3.203 (PMC5946158; doi:10.1002/iid3.203)
Supplement: Supplementary file 1 — Table S1. Monoclonal antibodies used in this study. [file IID3-6-194-s001.docx]

**Supplementary information**

**Table S1.** Monoclonal antibodies used in this study.

| **Target** | **Fluorochrome** | **Clone** | **Company** |
| --- | --- | --- | --- |
| CD3 | Brilliant Violet 510 | HIE3a | BD Pharmingen |
| CD3 | FITC | UCHT1 | Southern Biotech |
| CD4 | PE-Cy7 | RPA-T4 | Biolegend |
| CD4 | APC-Cy7 | OKT4 | Biolegend |
| CD8 | AmCyan | SK1 | BD Pharmingen |
| CD8 | PE Texas Red | 3B5 | Invitrogen |
| TNF | Alexa Fluor 700 | Mab11 | BD Pharmingen |
| IFNγ | [eFluor® 660](http://www.ebioscience.com/human-cd107a-antibody-efluor-660-ebioh4a3.htm) | 45·B3 | eBioscience |
| IL2 | eFluor450 | MQ1-17H12 | eBioscience |
| CX3CR1 | PE-Cy7 | 2A9-1 | Biolegend |
| CXCR3 | PerCP | 49801 | R&D |
| CCR5 | Pacific Blue | [J418F1](http://www.biolegend.com/index.php?page=pro_sub_cat&action=search_clone&criteria=J418F1) | Biolegend |
